# Natural Waxes as Gelators in Edible Structured Oil Systems: A Review

**DOI:** 10.3390/gels11080656

**Published:** 2025-08-18

**Authors:** Dafni Dimakopoulou-Papazoglou, Konstantina Zampouni, Eugenios Katsanidis

**Affiliations:** Department of Food Science and Technology, School of Agriculture, Faculty of Agriculture, Forestry and Natural Environment, Aristotle University of Thessaloniki, 54124 Thessaloniki, Greece; ddimakop@agro.auth.gr (D.D.-P.); zampounikona@gmail.com (K.Z.)

**Keywords:** oleogels, bigels, beeswax, carnauba wax, candelilla wax, rice bran wax, sunflower wax

## Abstract

The use of natural waxes to create edible structured oil systems, namely oleogels and bigels, represents an innovative approach to replacing trans and saturated fats in food products, offering healthier alternatives for the food industry. This review aims to provide a detailed overview of the utilization of natural waxes in the formulation of oleogels and bigels, their interactions with other ingredients, and the methods employed to assess their physicochemical properties. A comprehensive analysis is also presented on the impact of processing parameters on the physicochemical and structural characteristics of these systems, as well as their oxidative stability. Additionally, the application of structured oil systems in various food products, including spreads, dairy, and meat products, is explored, along with a discussion of the attributes of the final products.

## 1. Introduction

Fats and oils represent an important nutrient group, serving as a source of energy, enhancing flavor and texture of foods, and acting as carriers of essential nutrients and bioactive compounds. Additionally, in the food industry, fats (particularly solid fats) are necessary ingredients in a wide range of products as they contribute to the development of key properties such as structure, texture, flavor, aroma, and mouthfeel. Additionally, solid fats confer specific functionalities in various foods, such as meat or bakery products [1]. However, these fats are predominantly composed of saturated fatty acids and may contain significant amounts of *trans* fatty acids, whose increased consumption is linked to a higher risk of various diseases, including cardiovascular conditions [2,3]. Therefore, the Food and Agriculture Organization of the United Nations (FAO) and the World Health Organization (WHO) strongly emphasize the recommendation to reduce the consumption of saturated fatty acids and trans to less than 10% and 1% of total energy intake, respectively [2,4]. At the same time, growing consumer demand for foods with reduced fat content and improved nutritional profiles has driven the food industry to investigate alternative approaches. Nevertheless, the development of products with substantially reduced fat poses challenges related to structural integrity, safety, and sensory attributes, creating a significant hurdle in the field of food technology.

The search for and development of alternative approaches to fat replacement has been gaining considerable momentum in recent years, with a primary focus on the use of edible vegetable oils [5]. Edible structured oil systems mainly refer to systems in which an oil has been entrapped within a three-dimensional network through the use of gelators at low concentrations. This process, known as oleogelation, has been extensively studied over the past years using various gelators and edible oils (as highlighted in several reviews [1,6,7]). Additionally, recent research interest has shifted toward the formation of bigels, which are biphasic systems composed of a hydrogel and an oleogel phase. The use of oleogels and bigels as fat substitutes lies in their ability to enhance the nutritional profile of food products, and, in the case of bigels, by lowering overall fat intake. Oleogels consist of oil structured within a gel network, while in bigels both an aqueous phase and a structured oil phase coexist, thus reducing fat content. Various structuring agents have been successfully used to develop oleogels and bigels, including mono- and diglycerides, fatty alcohols, fatty acids, and phytosterols [8,9]. Notably, natural waxes have been gaining increasing attention in recent years as effective gelators in these structured oil systems [10,11,12].

Waxes are a diverse class of natural esters primarily formed by the reaction of long-chain fatty acids with long-chain alcohols, resulting in molecules characterized by extended hydrocarbon chains. This unique composition imparts a highly hydrophobic nature to waxes, making them insoluble in water and ideal for forming moisture barriers. Plants produce waxes to coat their leaves and fruits, protecting them from moisture loss and microbial attacks. Waxes are also produced by animals, such as beeswax, which is secreted by honeybees to construct honeycombs. In general, waxes consist of fatty alcohols, fatty acids, and hydrocarbon chains, with the ratios of these components depending on their biological source (Table 1). This composition directly influences their gelling behavior in various systems. The advantages of using natural waxes in oleogels and bigels include their ability to form gels at low concentrations, their good structuring properties, wide availability, and low cost [5,13]. For potential application in edible oleogels and bigels, various plant-derived waxes such as candelilla (CDW), carnauba (CBW), rice bran (RBW), and sunflower (SW) waxes have been investigated, along with animal-derived beeswax (BW). Certain waxes, including CDW (21 Code of Federal Regulations (CFR 175.105 and 184.1976), CBW (21 CFR 184.1978), RBW (21 CFR 172.890), and BW (21 CFR 184.1330), have been characterized as GRAS (Generally Recognized as Safe) by the FDA, allowing their use in foods for specified purposes under appropriate conditions. The main characteristics of these waxes are summarized in Table 1.

BW is a natural wax produced by honeybees (*Apis mellifera*) and is one of the most widely studied and utilized animal-derived waxes in edible applications. Chemically, BW is composed mainly of wax esters (mainly C16, C34–C54) formed from long-chain fatty acids and alcohols, along with hydrocarbons (C23–C31), and free fatty acids (C16, C24–C34) [5,17]. It has a melting point typically between 61 and 65 °C, offering moderate thermal stability that makes it suitable for structuring edible oils.

CBW is a hard, plant-derived wax obtained from the leaves of the Brazilian palm tree *Copernicia prunifera*. It is mainly composed of wax esters C56–C60), long-chain fatty alcohols (C28–C32), fatty acids (C24–C30), and hydrocarbons [16,17]. Among commonly used natural waxes, CBW exhibits the highest melting point, typically ranging from 80 to 85 °C, which contributes to its exceptional thermal stability.

CDW is extracted from the surface of the leaves and stems of the plants of *Euphorbia cerifera* and *Euphorbia antisyphilitica*, which are native to northern Mexico and the southern United States [17]. It is characterized by a high content of hydrocarbons (C26–C33), along with wax esters (C39, C41), free fatty alcohols and sterols (mainly C30), free fatty acids (C16–22), and moisture [14,17]. CDW can form stable gels at concentration as low as 1.5% [19], and its melting point ranges between 68 and 73 °C.

RBW is a natural wax obtained from the dewaxing of rice bran oil during its re-fining process. It mainly consists of wax esters (C44–C64), which comprises esters of saturated fatty acids (C16–C32) and fatty alcohols (C24–C8), with only low levels of free fatty acids and trace amounts of free fatty alcohols and hydrocarbons [18,20]. It has a relatively high melting point, around 78–82 °C, which provides excellent thermal stability and crystallization characteristics to form stable gel networks.

SW is derived from the hulls of sunflower seeds (*Helianthus annuus*) and is obtained as a by-product from the dewaxing process of sunflower oil refining. It mainly consists of wax esters (96–97%) of long-chain saturated fatty acids (mainly C20–22) and alcohols, while free fatty acids (C16–22) are present in low proportions (3%) [5,13]. Its melting point ranges between 74 and 77 °C, attributed to its high wax ester content, which supports stable gel formation [21].

The aim of this review is to provide a comprehensive examination of the use of natural waxes as gelators in the development of edible structured oil systems, with a particular focus on oleogels and bigels. Given the growing scientific interest in employing waxes for structuring edible oils, this review compiles and analyzes all relevant studies published from 2018 up to May 2025 that investigate the application of waxes in oleogels and bigels, specifically in food-related context. By offering a clear and up-to-date overview of recent advancements, this work aims to support the design of healthier, more stable, and consumer-acceptable fat replacers and delivery systems.

## 2. Wax-Based Oleogels

### 2.1. Definition and Structure of Oleogels

Wax-based oleogels are structured oil systems in which liquid edible oils are immobilized within a three-dimensional network formed by low concentrations of natural waxes. The gelation process occurs primarily on the self-assembly of wax molecules upon cooling, leading to the formation of crystalline networks that physically entrap and stabilize the edible oil. These systems have gained attention in the food industry as promising alternatives to conventional solid fats, due to their ability to deliver desirable textural and structural properties while reducing saturated and *trans* fatty acids content in food products [22,23]. Additionally, wax-based oleogels have also been explored as carriers for bioactive compounds, enhancing the nutritional and functional profile of food systems [24].

The characteristics of wax-based oleogels are largely determined by the type and concentration of the wax used, the presence and concentration level of other structuring agents, the composition of the oil phase, and various processing parameters such as mixing time and temperature, cooling rate, and stirring speed. These factors influence the crystalline morphology, gel strength, oil-binding capacity, and melting behavior of the oleogels. Different waxes exhibit distinct crystalline structures, spatial distributions, and van der Waals interactions, which in turn affect the properties of oleogels in unique ways [25,26]. Consequently, it becomes evident that oleogels with tailored chemical and physical properties can be developed by combining specific waxes with oils, thereby enabling their use in targeted food applications.

Recent studies have demonstrated the applicability of oleogels in a wide range of food products, including spreads, bakery items, meat products, and dairy formulations, highlighting their potential to replace traditional saturated fats without compromising desirable sensory and functional attributes. At the same time, research focusing on the use of natural waxes as oleogelators has grown rapidly in recent years. Therefore, the following section provides a comprehensive overview of all studies on wax-based oleogels published from 2018 up to May 2025, with particular emphasis on their formulations, properties, and applications.

### 2.2. Composition Design of Wax-Based Oleogels

The design of wax-based oleogels primarily involves selecting the appropriate type and concentration of wax, the choice of edible oil, and optimizing processing parameters to achieve the desired gel properties. Natural waxes such BW, CBW, CDW, RBW, and SW have been widely studied as structuring agents due to their strong ability to form crystalline networks at relatively low concentrations, with critical concentrations typically ranging from 1% to 4%, depending on the type of wax [13,27].

**Figure 1:**
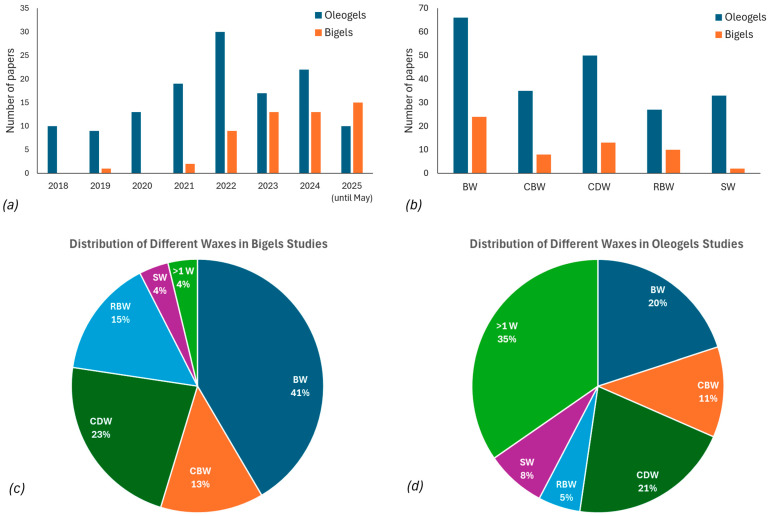
Recent studies related to the use of various waxes for the formation of oleogels and hybrid gels. (**a**) Distribution of studies reporting the use of wax-based oleogels and bigels in recent years. (**b**) Distribution of studies based on the type of wax used. (**c**) Distribution of different waxes in bigel studies. (**d**) Distribution of different waxes in oleogel studies.

In recent years, there has been growing interest in investigating the use of natural waxes for the formation of oleogels, with most researchers focusing on BW and CDW, accounting for approximately 50% of the total oleogel studies. When also considering studies that investigate systems with combinations of more than one wax (category > 1 W), BW appears in 66 out of 130 studies and CDW in 50 out of 130 studies. This is followed by CBW and SW, representing 11.5% (35/130 studies) and 7.7% (33/130 studies), respectively. Although RBW is less frequently studied on its own, it appears more often when combined with other waxes, highlighting its role as a complementary structuring agent (Figure 1).

The wax concentration used in most studies is relatively low (<10%), with many researchers evaluating levels around 5–6%. More specifically, the concentration ranges reported in the literature are approximately 3–11% for BW, 3–15% for CBW, 3–20% for CDW, 2–25% for RBW, and 3–12% for SW (Table 2). When different waxes are combined with each other or with other components, the total structurant concentration is generally similar or even lower (Table 2). Moreover, in order to reduce the required wax concentration while still achieving desirable textural properties and thermal stability, researchers frequently combine waxes with co-structuring agents such as glycerol monostearate (GMS) [28,29,30,31,32], monoglycerides (MGs) [26,33,34,35,36,37,38,39,40], stearic acid [41,42], lecithin [31,43,44], or synthetic surfactants like Span-60, Span-80, and Tween 80 [45,46]. This synergistic structuring approach enables the formation of stable gels at reduced wax levels, thereby minimizing waxy mouthfeel and lowering costs. Additionally, the incorporation of bioactive compounds such as thyme [47], cumin [42,47], quercetin [48], ascorbic acid [49], α-tocopherol [49,50], β-carotene [30,51,52], and resveratrol [53] has been explored, utilizing the oleogel matrix for controlled delivery and enhanced functional value.

#### Edible Oils

The choice of edible oil also plays a crucial role, as it impacts the nutritional profile, oxidative stability, and mouthfeel of the final oleogel. Sunflower oil is among the most commonly employed due to its neutral flavor and availability [30,41,44,45,54,55,56,57,58,59]. Studies have also explored the use of soybean oil [53,60,61,62,63,64,65,66,67,68,69], canola oil [32,34,49,50,52,70,71,72,73,74,75], rice bran oil [18,43,76,77], olive oil [47,78,79], safflower oil [80,81,82], corn oil [83,84], rapeseed oil [33,85,86,87], and hemp seed oil [88,89,90], aiming to enhance both physicochemical and nutritional properties of oleogels. In addition, less common oils such as flaxseed oil [28], grapeseed oil [29], walnut oil [39], linseed oil [91,92], fish oil [93,94], chia seed oil [95], groundnut oil [42,96], sesame oil [97], peanut oil [98], and black cumin oil [99] have also been evaluated for oleogel formation. Additionally, various studies have specifically investigated how the type of oil influences the properties of oleogels, including their microstructural, textural, and thermal characteristics [26,51,57,100,101,102,103,104,105,106,107].

**Table 2:** Recent studies on wax-based oleogels for food applications.

Oleogelators	Edible Oil	Characteristics Studied and Application	Ref.
**Beeswax**			
BW (10%), hydrocolloid blend (sodium caseinate (3.15%), guar gum (0.5%), XG (0.22%)	Sunflower oil	SFC, RM, TA, DSC, CM (Margarine formulation replacing palm oil and partially hydrogenated palm olein)	[108]
BW (3, 8%)	Sunflower oil	RM, DSC, OBC, CM, Potato strip analyses (Sensory, Oil Uptake) (as frying medium for potato strips)	[56]
BW (5%) + β-carotene (0–0.4%)	Canola oil	CM, PLM, RM, TA, DSC, FTIR	[109]
BW (10%)	Sunflower oil + shortening	SFC, Products properties (Gluten-free cake formulation)	[110]
BW (8%)	Linseed oil	Sausages properties (Pork backfat replacement in frankfurters)	[92]
BW (6%)	Olive, linseed, fish, and sunflower oil	PLM, TA, OBC, DSC, FTIR	[101]
BW (11%), EC (11%)	Blend of olive, linseed and fish oil	CM, RM, FTIR, DSC, OS (TBARS), Pate properties (Pork backfat replacement in pork liver pâtés)	[111]
BW (11%), EC (11%)	Blend of olive, linseed and fish oil	PLM, TA, DSC, OS (TBARS), FAC, CM, Burger properties (Pork backfat replacement in low-fat pork burgers)	[102]
BW (4%)	Linseed, corn, sunflower, and camellia oil	FAC, OBC, PLM, RM, DSC, FTIR, XRD	[112]
BW + MGs (7, 10%) (1:2)	Sunflower oil	Semi-smoked sausages properties	[113]
BW (4%) + β-cyclodextrins	Corn and fish oil	SEM, TGA, FTIR, Particle size, ζ-potential, SE	[114]
BW (5%) + WPI coating	Fish oil	RM, SEM, Encapsulation efficiency, Particle size, ζ-potential, TBARS, Micropolarity, Microviscosity (OG for stabilization and delivery of ω3 in fish oil)	[93]
BW (3–5%)	Sunflower oil, Medium- and long-chain triglyceride, Diacylglycerol	FAC, MGC, PLM, OBC, TA, DSC, SFC, FTIR, XRD	[115]
BW (10%) + Ascorbic acid or α-tocopherol (0.01–0.03%)	Canola oil	OM, TA, RM, DSC, XRD, FTIR, OBC, PV, OS (p-AV, TOTOX)	[49]
BW (5–10%)	Sesame oil	FAC, CM, PLM, TA, DSC, Product properties (substitute of animal fat for beef burgers)	[97]
BW + SHW (10%) (70:30)	Canola and linseed oil	TA, OBC, RM, DSC, CLSM, FAC, OS (PV)	[116]
BW, β-sito blends (10%)	Sunflower oil	PLM, CM, TA, DSC, FTIR, XRD	[117]
BW (3–4%)	Camellia, soybean, sunflower, and flaxseed oil	PLM, DSC, FTIR, XRD, PV (Comparison of oil types in BW-oleogel formation)	[57]
BW (8–9%)	Avocado, sunflower, and linseed oil (with 0.2% curcumin)	TA, OBC, RM, OS (PV, K268), Curcumin degradation kinetics, In vitro digestion, FFA/curcumin bioaccessibility	[118]
white BW (3–11%)	Olive, grape seed, walnut, hemp seed, and sunflower oil	SEM, OBC, PV, CM, FTIR	[88]
BW (6%)—combinations of hydrocarbons, monoesters, di-/triesters + FFAs + FAl (C)	Sunflower oil	PLM, TA, OBC, DSC	[119]
BW and BW hydrocarbons (6%)	Sunflower oil	PLM, TA, DSC (study under different cooling rates)	[58]
BW/StA (11.74%) (3:1) + β-sito (5%)	Sesame, rice bran oil, and blends	PLM, OBC, RM, DSC, FTIR, OS	[120]
BW (6%) and combinations of its fractions	Sunflower oil	OS (PV, AV, CDV, TOTOX), TA, FAC	[121]
BW (3%) or BW/hydrocarbon (9:1)	Sunflower oil	CM, TA, FAC, DSC, SE, Product properties (substitutes for solid fats in margarine)	[122]
BW (2–8%)	Peanut oil	OM, RM, TA, OS	[98]
**Carnauba wax**			
CBW (6–10%)	Soybean and peanut oil	PS, PLM, OBC, DSC, SS, SE (fat replacer for ice cream)	[123]
CBW (4–8%) and Propolis wax (5–10%)	Safflower oil	PS, OBC, SFC, CM, FAC, PV, TPA, SE (fat substitutes in cake batters)	[80]
Different types of CBW (4–8%)	Soybean oil	PS, PLM, CE, TA, RM	[60]
CBW (5–15%)	Soybean oil	TA, OBC, SP, Product properties (as frying medium for Indian traditional snack (Mathri))	[61]
CBW + Adipic acid (6%)	Soybean oil	PLM, CM, RM, FTIR, XRD, DSC, OBC, OS, Products properties (fat substitutes in cake and beef burger)	[62]
CBW (3–9%)	Canola oil	SFC, Product properties (fat substitutes in imitation cheese)	[70]
CBW + MGs (5–10%)	Canola oil	RM, DSC	[34]
CBW, β-sito/lecithin, EC, resveratrol (combinations)	Soybean and peanut oil	PLM, TPA, RM, DSC, FTIR, XRD, CM, OBC, SFC, in vitro, determination of bioavailability	[53]
CBW (6%)	Sunflower and linseed oil in various ratios	FAC, PLM, CM, OBC, DSC, FTIR, XRD, OS, Product properties (shortening substitution in cakes)	[124]
CBW (6%) and CBW + Adipic acid (6%)	Sunflower oil	Product properties (fat substitutes in chocolate spread)	[54]
CBW (8%)	Soybean oil	OBC, TPA, RM, DSC, FTIR, SP (optimize the ultrasonication conditions)	[63]
CBW (6%)	Acorn and soybean oil	PLM, CLSM, RM, TPA, DSC, XRD, FTIR, OBC, PS, Product properties (Chocolate spreads preparation)	[125]
CBW (10%)	Sunflower oil	TPA, RM, Products properties (fat replacer in pastries (bow tie cookies, cheese crackers, apple pie, cookies, jam-filled puff pastry))	[126]
CBW (5–15%)	Soybean oil	OBC, TPA, RM, DSC, FTIR, SP	[64]
Monopalmitate + CBW (10%)	Soybean oil	OBC, SFC, DSC, NMR	[65]
**Candelilla wax**			
CDW (5%)	Canola oil	Products properties (Preparation of cake (blends of canola oil oleogel/butter))	[71]
CDW (1–5%) + StA (0.005–0.05%), curcumin (5%)	Groundnut oil	PLM, MGC, OBC, CM, FTIR, Raman, XRD, TA, in vitro curcumin release	[42]
CDW (5%) + StA (0.015%)	Groundnut oil	Product properties (different pasta samples with OG)	[127]
CDW (5%) + olive diacylglycerol stearin (5–35%)	Olive triacylglycerol oil	FAC, PLM, OS (PV, TBA), Product properties (Substitution of margarine, cookies)	[128]
CDW + GMS (10%)	Grapeseed oil	PLM, BLM, DSC, RM, TA, OBC, NMR	[29]
CDW + MGs + fully hydrogenated oil (5–10%)	Soybean and high-oleic sunflower oils	PLM, TA, RM, OBC, DSC, SFC (NMR)	[35]
CDW + Hard fats (5%)	Soybean oil	PLM, FAC, DSC, RM, OBC, SFC (NMR)	[19]
CDW (3–9%)	Extra-virgin linseed oil	CM, MP, TA, FTIR, Product properties (Replace fat in cookies)	[91]
CDW (10%), CDW + GMS (1:3), β-carotene	Sunflower oil	TA, RM, OS (PV), Product properties (applications to muffin as a shortening replacer)	[30]
CDW (5%)	Groundnut Oil	Product properties (substituting water with oleogel in pasta)	[96]
CDW + GMS (10%)	Canola oil	TA, DSC, Product properties (shortening replacer in filling creams)	[32]
CDW (10%) + quercetin (0.02–0.06%)	Sunflower oil	OM, FTIR, XRD, OBC, RM, CM, OS (PV), Products properties (Replace fat in meat batter and sausages)	[48]
CDW (3%), β-carotene	Peanut, pine nut and walnut oil	PLM, RM, TA, XRD, OBC, Product properties (β-carotene encapsulation)	[51]
CDW (3%)	Canola oil	Product properties (Replace solid saturated fat in sponge cake bread)	[72]
CDW (10–20%) + phosphorus (0–3%)	Safflower oil	RM, OBC, DSC, Evaluation of the phosphorus release	[81]
CDW (0.75–4%) + α-tocopherol (0.5–10%)	Canola oil	PLM, OBC, RM, TPA, DSC, NMR	[50]
CDW (1–8%) + MGs (0.35–0.7) or polyglycerol polyricinoleate (0.25–0.5)	High oleic safflower oil	RM, DSC, XRD, NMR	[36]
CDW + MGs (10%)	Walnut oil	PLM, TPA, RM, FTIR, Product properties (Replace butter in chocolate spreads)	[39]
CDW (0–3%), EC (0 -12%), MGs (0, 5%)	High oleic safflower oil	PLM, DSC, RM (compared with fat phase of stick, Danish, and puff pastry margarines)	[129]
CDW (3, 9%)	Hemp seed and olive oil	OBC, OS (PV), CM, Product properties [plant-based ice creams (oat milk, millet milk and spelt milk, sugar, oleogel and flavors)]	[88]
CDW (5%) + lecithin from sunflower and soya	Rice bran oil	PLM, CM, Surface Topology, FTIR, DSC, TA	[43]
CDW (3, 8%) and MGs (0.7%) or PGPR (0.5%)	High oleic safflower oil	PLM, SEM, TEM, XRD	[37]
CDW (3%) + flaxseed gum (0–0.4%)	Flaxseed oil	TA, OBC, DSC, RM, XRD	[130]
CDW (3–8%)	Rapeseed and linseed oil (1:1)	CM, PM, PLM, RM, PS	[131]
CDW or GMS (10%)	Sunflower oil	TPA, OBC, Product properties (Replace fat in Bologna Sausages)	[55]
CDW (3%)	Chia seed oil	OM, TA, RM, XRD	[95]
CDW (5%)	Canola oil	Product properties (preparation of maize tortillas)	[73]
**Rice bran wax**			
RBW (2–10%)	Corn oil	PLM, TA, SFC, DSC, XRD, in vitro	[83]
RBW (3, 7%)	Olive, sunflower, flaxseed, soybean, and medium-chain triglyceride (MCT) oil	MGC, TA, OBC, PLM, DSC, OS	[132]
RBW (0.5–5%)	Sunflower oil	DSC, OBC, XRD, FTIR	[59]
RBW (2.5 or 10%)	Conventional and high-oleic soybean oil	Product properties (alternatives to pork fat in chicken-based bologna sausage)	[133]
RBW (1–11%)	Rice bran oil	TA, SFC, XRD, PLM, DSC, OS	[134]
RBW (0.5–25%)	Rice bran oil	WC (HPLC), DSC, PLM, RM	[18]
RBW (2.5 and 10%)	Soybean oil	Product properties (replace pork fat in frankfurter-type sausages)	[66]
**Sunflower wax**			
SW, BEW, GMS, different ratios (6%)	Flaxseed oil	PLM, SEM, RM, DSC, OBC, OS,	[28]
SW (5%), Span-80 and Tween-80 (1–10 mg)	Sunflower oil	OBC, CM, PLM, DSC, FTIR, XRD, Spreadability study, curcumin release	[135]
SW (5%), Span-60 and stearyl alcohol (1–10 mg)	Sunflower oil	OBC, CM, PLM, TA, DSC, FTIR, XRD, curcumin release	[46]
SW (5%), Span-80, Span-60, Tween-80, and stearyl alcohol (0.05–0.015%)	Sunflower oil	FAC, CLSM, DSC, Raman, Properties of probiotic (as growth modulator of probiotics)	[45]
SW, SW + MGs (6–12%)	Olive, sunflower, sesame, and soybean oil	CM, PLM, TA, DSC, FTIR, OS	[136]
SW (3–7%)	Soybean oil from 3 types of seeds	PLM, RM, TA, DSC, SFC	[137]
SW (3, 7%)	Olive, canola, corn, soybean, grapeseed, sacha inchi, chia seed, and flaxseed oil	FAC, TocA, TPCA, FFA, TA, DSC, WC	[100]
SW (5%), MGs (5%)	Rapeseed oil	TA, Product properties (as frying medium for French fries)	[85]
SW, EC, and MGs individual or in mixtures (5–10%)	Rapeseed oil	OBC, TA, SEM, RA, Product properties (Cookie Preparation)	[33]
SW (10%) + thyme and cumin (1%)	Virgin olive oil	OBC, SFC, CM, FFA, XRD, DSC, RM, Volatile Compound Analysis, SE, Consumer Tests	[47]
**Different/combined waxes**			
BW, CBW, SW (6–10%)	Moringa, tiger nut and garden cress oil	OBC, Total phenolic content, FAC, OS, TPA, CM, DSC	[104]
BW, CBW, SHW, SW, MGs (7–14%)	Laurel oil	TA, PLM, XRD, TGA, NIR	[38]
BW, RBW (3–10%)	Safflower oil	OBC, SFC, CTD, CM, FAC, OS (PV, FFA, K232, K270), Product properties (shortening replacers in cakes)	[82]
BW (4–8%), CBW (4–8%)	Pumpkin seed oil, sunflower oil (for comparison)	DSC, RM, TA, SFC, OBC	[138]
BW, CBW (6%)	Pumpkin, hemp, almond, rice, sesame, and grapeseed oil	FAC, DSC, RM, SFC, OBC, CM	[105]
SW, BW (1–15%)	Eucalyptus, lavender, lemon peel and tea tree essential oils	OBC, OS, CM, TA, XRD, DSC, TGA	[139]
SW, BW (5–15%)	Black cumin seed, St. John’s Wort, and grape seed oils	OBC, CM, TA, XRD, DSC, Volatile composition	[107]
RBW, CDW, SW, and BW together with MGs (tot. conc. 15%)	Olive, sunflower, sesame, and soybean oil	CM, PLM, TA, MP, FTIR	[26]
BW, RBW, SW, StA, Octadecanol, γ-β, and EC (10%)	Sunflower oil	PS, CTD, DSC, PLM, OBC, RM	[41]
RAW, RBW, SW, BW, MGs, γ-β (5–15%)	Medium-chain triacylglycerides oil	PS, PLM, OBC, RM	[40]
SW, RBW, CDW, and BW (2–4%) various binary wax blends (1:1, 1:3, and 3:1 *w*/*w*)	Olive oil	DSC, TA, PLM, OBC, FAC	[78]
CDW (3, 7%) or RBW (5, 7%)	Hemp oil	SFC, FFA, FAC, OS (PV, CDV, TBARS), Product properties (Replace animal fat in meat patties)	[89]
CDW + BW (3–7%)	Soybean oil	TA, DSC, SFC, Product properties (Margarine formulation)	[67]
SW (1–1.5%), RBW (8–10%)	Sunflower, mustard, soybean, sesame, groundnut, rice bran, palm, and coconut oil	DSC, RM, SEM, XRD, CTD, OBC, SFC	[106]
RBW, SW (0.5–1%)	Soybean oil	DSC, Product properties (Replace solid milk fat in Swiss cheese)	[140]
BW (3%), RBW (1–9%), SW (1–9%), CW (3%)	Fish oil	OS (PV, CDV, eicosapentaenoic acid (EPA) and docosahexaenoic acid (DHA) levels), DSC, NMR, TA, CM	[137]
RBW, SW, CDW, BW (3–7%)	Cold-pressed hempseed oil	FAC, TA, SFC, OM, DSC, Product properties (fat replacement in margarines and spreads)	[141]
CDW, CBW, BW (10%)	Insect oil (from Tenebrio molitor larvae)	DSC, RM, TA, OBC, PV, OS, Product properties (shortening replacement in cookies)	[142]
SW, CDW, BW (white and yellow), MGs (5%)	Rapeseed oil	TA, OBC, CM, OS, OM, PLM	[86]
BW, CBW, CDW, RBW, sitosterol, pea protein, and XG, (9%)	Hemp seed oil	Dough and cookies properties (Replacing margarine in cookies)	[90]
CBW, BW, CDW, RBW (5%), MGs (7, 12%), and mixtures wax (1–5%) with MGs (7%)	Sunflower oil	MGC, PLM, TA, OBC, DSC, RM, FTIR, in vitro	[25]
SW, CBW, CDW, BW, BEW, FW + Soybean lecithin (LEC), various ratios FW:LEC (0–100%) (1.75–7%)	Sunflower oil	DSC, RM, PLM, SEM, Raman	[44]
White/Yellow BW (5%), CDW (2–3%), RBW (2%), EC (8%), MG (5%)	High-oleic rapeseed oil	TA, OBC, DSC (Short-dough biscuits reformulated to replace palm oil with OG)	[87]
BW (10%), SW (7%), CBW (7%)	black cumin oil	DSC, PLM, OBC, RM, TA, OS	[99]
CDW, BW, RBW, CBW (1–10%)	Rice bran oil	MGC, PLM, TA, XRD, Product properties (replace fat in cookies	[76]
SBW, BW, CBW, binary mixtures (0–9%)	Sunflower oil	TA, CM, SP, FAC, PLM, FTIR, XRD	[143]
CDW, BW, CBW, EC with diff. mass ratios (10%)	Corn oil	PS, TPA, OBC, CM, FTIR, DSC, RM, PLM	[84]
BW, RBW (5 and 9%)	Grape seed, hemp seed, olive, sunflower, and walnut oil	RM, DSC, TGA, TPA, Dough properties (Dough preparation using oleogels)	[144]
CDW, BW (6–12%)	Virgin coconut and mustard oils	OBC, CM, CLSM, RM, TPA, FTIR, SP	[145]
BW, CDW, SW, and RBW (4, 10%)	Canola and sunflower oil	Oil purification and increment of polar oil components, RM, TA, DSC, BLM	[146]
BW, CDW, CBW (0.5–5%)	Camellia oil and medium chain triglycerides	MGC, OBC, TA, DSC, XRD, PLM, FTIR	[147]
BW, CDW, CBW (1–6%)	Extra virgin olive oil	PCM, OS (PV), TA, DSC, RM	[79]
BW, CBW, CDW and RBW (5%)	Soybean oil	OBC, CTD, PLM, RM, Characteristics of chips (Deep-frying potato chips)	[68]
CBW, GMS, β-sito/BW (10%), and β-sito: lecithin (16%)	Sunflower oil	TA, OBC, RM, Product properties (tender dough products using oleogels)	[31]
BW (3%), CBW (6%), EC (4, 8%) and mixtures	Rice bran oil	PS, PLM, CLSM, RM, TA, OBC, DSC, XRD, FTIR	[77]
CBW + BW different ratios (4%)	Rice bran oil	PS, PLM, TA, RM, OBC, DSC, XRD, OS	[148]
CDW or CDW + BW (1:1) (3%)	Canola oil	RM, XRD, OBC (potential to mimic commercial margarine)	[74]
SW, BW, hydrolyzes SW and BW, combinations (ratios 0–100) (8, 12%)	Canola oil	Composition of waxes, BFM, SEM, DSC, RM, TA	[75]
SW, RBW, BW, CDW, SCW, and CBW (10%)	Canola oil and medium-chain triglycerides oil	DSC, BFM, PLM, TA, RM	[149]
BW, CDW, SW (5%) and binary mixtures	Soybean oil	DSC, phase contrast light microscopy, SFC, TA	[150]
BW, SW, CDW, RBW (0.5–2%)	Fully hydrogenated cottonseed oil	OBC, FAC, SEM, Properties of peanut butter (waxes as stabilizers in peanut butter)	[21]
BW + CDW + SW, different ratios (5%)	Soybean oil	WA (HPLC), MGC, XRD, DSC, TA, PLM, SFC, NMR, RM	[69]
Tea wax, RSW, orange peel wax, rose wax, and BEW, compared to SW (1–25%)	Sunflower oil	CTD, OBC, SFC, CM, PV, DSC, PLM, XRD, RM, SE	[151]
SW, BW (10%)	Flaxseed oil, Tallow fat (melted, filtered, and stored)	CM, FFA, DSC, Product properties (Production of the sucuk samples)	[152]
RBW, CW, BW, CBW (6%)	Rice bran oil	Rice cooking properties	[153]

BW: beeswax, CBW: carnauba wax, CDW: candelilla wax, RBW: rice bran wax, SW: sunflower wax, SHW: soybean wax, SHW: shellac wax, BEW: berry wax, FW: fruit wax, SCW: sugarcane wax, MGs: monoglycerides, GMS: glycerol monostearate EC: ethylcellulose, β-sito: β-sitosterol, γ-β: γ-oryzanol/β-sitosterol, StA: stearic acid, XG: xanthan gum, SL: soy lecithin, PS: physical stability, CM: color measurement, OM: optical microscopy, PLM: polarized light microscopy, CLSM: confocal laser scanning microscopy, SEM: cryogenic scanning electron microscopy, LSM: laser scanning microscope, BLM: bright-field microscopy, TEM: transmission electron microscopy, RM: rheology measurement, TA: texture analysis, DSC: differential scanning calorimetry, TGA: thermogravimetric analysis, FTIR: Fourier-transform infrared spectroscopy, NMR: nuclear magnetic resonance, XRD: X-ray diffraction, OBC: oil binding capacity, MGC: minimum gelling concentration, CTD: crystallization time determination, SP: smoke point, MP: melting point, SFC: solid fat content, FAC: fatty acid composition, FFA: free fatty acids analysis, SWC: solid wax content, TPCA: total polar compound analysis, TocA: tocopherol analysis, OS: oxidative stability, PV: peroxide values, TBA: determination of 2-thiobarbituric acid, p-AV: *p*-anisidine value, CDV: conjugated diene value, TOTOX: total oxidation value, SE: sensory evaluation, SS: storage stability.

### 2.3. Physicochemical and Structural Characterization of Oleogels

The evaluation of the properties of wax-based oleogels is carried out to assess their suitability and effectiveness across a wide range of applications, such as their use as fat substitutes. The properties typically studied include the microstructural characteristics, mechanical strength, viscoelastic behavior, and the oxidative and storage stability of the oleogels.

Specifically, the characterization of oleogel properties involves visual observation to determine the critical concentration required for gel formation, as well as color measurements. Additionally, microstructural analysis is performed using polarized light microscopy (PLM), which reveals the types of crystalline structures present. Rheological and textural properties are also evaluated through rheometry and texture analysis, assessing parameters such as hardness and cohesiveness of the system. Thermal properties are investigated using differential scanning calorimetry (DSC) and thermogravimetric analysis (TGA) to understand melting, crystallization, and thermal transitions. Furthermore, other physicochemical analyses, such as Fourier transform infrared spectroscopy (FTIR) and X-ray diffraction (XRD), are employed to explore molecular interactions and crystalline structures. Oil binding capacity (OBC), along with assessments of the structural and oxidative stability of the oleogel during storage, are also key factors for a comprehensive description of the system’s properties.

#### 2.3.1. Microstructural Analysis

The microstructural arrangement of oleogels largely determines their texture, oil-binding capacity, and overall stability. The choice of wax, the presence or absence of other structuring agents, and the type of oil used directly influence the morphology of the crystals and their spatial distribution [26]. PLM revealed that increasing CBW concentration (6–10%) led to larger mean crystal diameters and a denser crystal network in soybean and peanut oils oleogels. Specifically, at 10% CBW, large and organized crystalline networks were observed, which contributed to improved oil binding and gel stability [123]. Han et al. [112] observed that BW formed needle-like or platelet-shaped crystals in oleogels, with crystal aggregation increasing at slower cooling rates. Oils with higher levels of polyunsaturated fatty acids (PUFA), especially linseed oil, formed denser and more prevalent crystalline networks. The fractal dimension was significantly higher for linseed oil-based oleogels, indicating closer crystal packing and greater network complexity. Dent et al. [83] evaluated RBW oleogels (2–10% *w*/*w*) in corn oil for their potential as curcumin delivery systems, showing that higher RBW concentrations produced denser and larger crystalline networks, while the addition of curcumin did not change the crystal morphology. Microstructural analysis showed a transition from flocculent to long dendritic crystal structures as RBW concentration increased, forming denser three-dimensional networks at higher wax levels [134]. Similarly, Wijarnprecha et al. [18] examined the microstructural properties of RBW (0.5–25%) oleogels prepared with rice bran oil, suggesting that the increase in RBW concentration led to networks of interlinked, high aspect-ratio wax crystal needles (up to 50 μm), which became denser and more entangled at higher wax levels, facilitating superior oil binding and gel stability. Bharti et al. [135] stated that emulsifiers significantly affected crystal morphology; Span-80 increased branching and crystal size at lower concentrations, whereas Tween-80 promoted fibrous network formation. Further, Bharti et al. [46] demonstrated that emulsifiers such as Span-80 and stearyl alcohol promoted larger and denser wax crystal formation through co-crystallization, with higher emulsifier concentrations driving a transition from fine, fiber-like structures to longer, well-organized crystals. Additionally, SW alone formed needle-like crystals, while combinations of SW with MGs resulted in crystal aggregates and rosette-like structures. These microstructures were dynamic, evolving during storage and suggesting ongoing molecular interactions and structural transformations over time [136].

#### 2.3.2. Rheological and Structural Properties

Rheological studies consistently demonstrate the viscoelastic nature of oleogels, with network strength and elasticity highly dependent on formulation and environmental conditions. Barroso et al. [28] reported that flaxseed oil oleogels with SW, BEW (berry wax) or GMS at ratios up to 6% exhibited viscoelastic behavior, with stronger gel structures observed at 5 °C compared to 25 °C. Mixtures of GMS:SW improved network strength and elasticity, whereas GMS:BEW combinations weakened the gel network. Similarly, Hwang et al. [137] found that oleogels demonstrated the highest storage modulus (G′) at low temperatures, reflecting increased solid content. Interestingly, G′ did not directly correlate with gel firmness, suggesting these parameters reflect distinct structural aspects of the oleogels. In contrast, Wijarnprecha et al. [18] showed a clear relationship between RBW concentration and rheological properties. Both storage (G′) and loss (G″) moduli increased proportionally with RBW content, indicating the formation of progressively stiffer and more elastic gel networks. The critical gelling concentration was as low as 0.5%, while higher concentrations (>10%) produced robust, self-supporting oleogels with markedly enhanced rheological strength. Storage (G′) and loss (G″) moduli increased with BW concentration and were highest in linseed oil oleogels, indicating stronger and more elastic networks. The interfacial tension of crystal melting (δ) was lowest in linseed oil oleogels, supporting enhanced network stability due to reduced interfacial energy [112].

Regarding hardness, it is generally observed that as the concentration of structuring components increases, so does the hardness of the system [31,104]. This is because wax molecules form a denser crystalline network, thereby reinforcing the gel matrix. Ingredients such as MGs, when combined with waxes, can further enhance hardness. Dimakopoulou-Papazoglou et al. [26] observed that when CDW and SW were combined with MGs at a 1:1 ratio at the same total concentration (15% *w*/*w*), the system’s hardness increased, whereas no such differences were noted for BW and RBW. On the contrary, da Silva et al. [35] did not detect any significant differences in hardness for oleogels containing combinations of CDW, MG, and hard fat (at total concentrations of 5–10% *w*/*w*) in soybean oil and high oleic sunflower oil. Apart from the critical concentration, which plays a key role in determining the structure and thus the hardness of oleogels, another important factor is crystal size. According to Scharfe et al. [146], the hardness of oleogels made with canola oil at 10% wax followed the order SW > CDW > RBW > BW. In contrast, Dimakopoulou-Papazoglou et al. [26] reported the order as SW > BW > CDW > RBW in oleogels of different oils at a concentration level of 15%. Wettlaufer et al. [149] observed that for 10% *w*/*w* wax oleogels in canola oil, the hardness order was SW > sugarcane wax > CDW > CBW = BW = RBW, while in medium chain triglycerides it was SW > RBW > sugarcane wax > CBW > CDW. Similarly, Pang et al. [76] noted that at critical concentrations, the hardness order was CBW > RBW > CDW > BW.

#### 2.3.3. Thermal Properties

Thermal analysis plays a crucial role in the characterization of oleogels. Differential scanning calorimetry (DSC) studies demonstrate how the added structurants influence the thermal behavior of the oleogels. SW-based oleogels typically displayed a single melting peak between 60 and 64 °C, indicative of stable crystalline structures [47,135,136]. The incorporation of emulsifiers significantly altered thermal transitions; Bharti et al. [46] reported slower crystallization rates and higher melting enthalpies (ΔH) in SW oleogels containing emulsifiers, suggesting enhanced thermal stability and polymorphic transitions to stable crystal forms. Similarly, Dimakopoulou-Papazoglou et al. [136] found that SW plus MGs oleogels exhibited multiple endothermic peaks, characteristic of complex polymorphism (α-, sub-α, and β-crystals) due to the presence of MGs. The reduced ΔH in SW/MG oleogels implied lower energy requirements for the network formation at higher concentrations. Hwang et al. [137] demonstrated that SW crystals facilitated earlier crystallization of fats, reflected by slightly higher onset temperatures, although wax itself had minimal influence on the melting/crystallization behavior of the soybean oil from three types of seeds. Crystallization enthalpy measurements confirmed greater solid formation in high-stearic oils during cooling.

Airoldi et al. [123] reported melting peaks in CBW oleogels between 79 and 82 °C, attributed to CBW esters and fatty alcohols. The ΔH increased by approximately 30% as CBW concentration increased, reflecting greater crystalline quantity and stronger network formation. Second heating cycles revealed the co-crystallization between CBW and soybean or peanut oils, with slight shifts in melting temperatures, indicating more integrated crystal networks. Roufegarinejad et al. [54] also observed that increasing linseed oil proportion slightly decreased the melting onset and peak temperatures, due to the effect of unsaturated fatty acids on molecular packing; however, CBW maintained sufficient crystalline structures for oleogel stability even in linseed oil-rich formulations.

In BW-based oleogels, Han et al. [112] reported that formulations with oils high in PUFA, particularly linseed oil, exhibited high melting and crystallization temperatures and enthalpies, implying enhanced thermal stability and dense crystal networks. Similarly, Morales et al. [116] observed that a mixture of BW and shellac wax (70:30 *w*/*w*, 10%) in canola and linseed oils displayed two distinct melting and crystallization peaks, typical of dual wax systems.

RBW oleogels consistently showed single, sharp melting and crystallization peaks across studies. Wang et al. [134] reported increasing ΔH with higher RBW content, indicative of enhanced crystallinity and network stability. Wijarnprecha et al. [18] similarly observed proportional increases in transition temperatures and ΔH with RBW concentration and noted excellent thermoreversibility during repeated heating and cooling cycles. Dent et al. [83] confirmed the above findings and further demonstrated that curcumin addition (0.1% *w*/*w*) did not significantly alter the thermal properties of RBW oleogels, suggesting compatibility of bioactive incorporation without compromising structural integrity.

#### 2.3.4. FTIR Analysis and X-Ray Diffraction

Fourier transform infrared spectroscopy (FTIR) is widely employed in the analysis of oleogels to provide insights into their molecular interactions and structural characteristics. By detecting the vibrational modes of functional groups, FTIR allows for the identification of specific chemical bonds and the evaluation of potential interactions between the constituents of the oleogel, such as waxes, oils, and any added co-structuring or bioactive compounds. This technique is particularly useful for detecting shifts or changes in characteristic absorption peaks, which may indicate the formation of new interactions, such as hydrogen bonding or van der Waals forces, between the oil phase and the gelators. Bharti et al. [135] reported no significant changes in the chemical structure of SW oleogels upon the addition of Span-80 and Tween-80, suggesting that emulsifiers were physically incorporated into the wax network without forming new chemical bonds. In contrast, Dimakopoulou-Papazoglou et al. [26] observed that systems containing MGs exhibited stronger hydrogen bonding and van der Waals interactions compared to oleogels structured with waxes alone (BW, CDW, RBW, and SW), as evidenced by characteristic FTIR peaks. Han et al. [112] reported van der Waals forces as the dominant interaction in all BW oleogels with various oils, with minor shifts in methylene and carbonyl stretching bands reflecting indirect differences in oil–BW interactions influenced by the type of oil used.

X-ray diffraction (XRD) is another essential analytical technique used to investigate oleogels, offering insights into their crystalline structure and polymorphism. Through diffraction patterns, XRD provides detailed information on the arrangement and spacing of crystalline lattices formed by wax molecules within the oleogel matrix. Han et al. [112] reported that XRD confirmed β′-type polymorphs across all formulations, with BW linseed oil-based oleogels showing the highest crystallinity, followed by corn oil and sunflower oil and the lowest in camellia oil. XRD analyses have consistently demonstrated the critical role of emulsifiers in influencing oleogel crystallinity and polymorphism. Bharti et al. [135] reported that oleogels exhibited β′ polymorphs, which are considered desirable for achieving optimal spreadability and texture. The addition of emulsifiers enhanced peak intensity and crystallite size, suggesting improved lateral packing of crystals and greater structural stability. XRD also confirmed the presence of β′ polymorphs, desirable for food applications, and indicated that curcumin remained solubilized within the oleogel matrix without recrystallization [83].

#### 2.3.5. Oil Binding Capacity

Oil binding capacity (OBC) is a critical parameter for evaluating oleogels, as it reflects the ability of the gel network to immobilize and retain liquid oil within its structure under various stress or storage conditions. Airoldi et al. [123] reported that OBC significantly improved at higher CBW concentrations (8% and 10%), achieving higher oil retention compared to 6% CBW formulations. Similarly, Alshehri et al. [104] reached the same conclusion when examining BW, CBW, and SW, observing that increasing the wax concentration from 6% to 10% generally led to higher OBC, except in the case of tiger nut oil with 6% CBW. This exception suggests that the high oleic acid content (69.55%) of tiger nut oil may result in a weaker gel structure due to lower interactions between monounsaturated fats and the crystalline wax network. Bharti et al. [135] investigated the effect of emulsifiers (Span-80 and Tween-80) on SW oleogels prepared with sunflower oil. The study reported that oleogels formed self-standing structures with excellent oil binding capacity (>99%) even with emulsifier addition. Also, Bharti et al. [46] evaluated the effect of other emulsifiers (sorbitan monostearate and stearyl alcohol) on SW-based oleogels. All formulations with 5% SW in sunflower oil exhibited excellent oil binding capacity (>99%), and emulsifier addition did not significantly modify oil retaining.

#### 2.3.6. Oxidative Stability

One of the important attributes evaluated for the application of oleogels in various food products is their oxidative stability, as it directly affects the sensorial characteristics of the products during processing and storage, specifically when oleogels are used as fat replacers. Lipid oxidation is affected by the type of gelling agent, the oil used, as well as storage stability and the presence of antioxidant compounds. The extent of lipid oxidation is typically determined by measuring primary oxidation products, such as peroxides (PV), and secondary oxidation products, like TBARS.

Studies on oxidative stability have shown that oleogels can effectively preserve oil quality over storage. Barroso et al. [28] reported that oleogels maintained oxidative stability comparable to liquid flaxseed oil over 30 days, with PV remaining within acceptable limits. Additionally, PV of oleogels containing 9% crude RBW increased only slightly over 90 days at 20 °C, outperforming oleogels made from refined wax [134]. Dimakopoulou-Papazoglou et al. [136] found that both SW and SW + MGs oleogels exhibited excellent oxidative stability over 28 days at 25 °C and 35 °C, with PV and TBARS values consistently below acceptable thresholds and significantly lower than those of liquid olive oil. Oleogels formulated with CBW (6–10%) exhibited excellent stability over 60 days at both 5 °C and 25 °C, showing better oxidative stability compared to pure oils [123]. Notably, a CBW oleogel composed of 50% sunflower oil and 50% linseed oil, provided a balance between oxidative stability and nutritional benefits [54]. Morales et al. [116] further confirmed the oxidative stability of BW/shellac wax oleogels in canola and linseed oils. PV remained within standards throughout storage, although Rancimat analysis indicated slightly lower oxidative stability compared to commercial fats.

## 3. Wax-Based Bigels

### 3.1. Definition and Structure of Bigels

Bigels are biphasic systems resulting from the combination of two different types of gels, specifically a hydrogel and an oleogel [154]. Bigels differ from other biphasic systems in that both phases are structured, and therefore, the use of an emulsifier is not required for their formation [9]. Since the system contains two distinct phases, there are many possible combinations of gelators that can be used in a bigel (Table 3). Bigels have been primarily studied in the field of pharmaceutical applications; however, in recent years, there has been growing interest in their use in food systems, particularly as carriers of bioactive compounds and as structured fats with improved functional properties [9,155,156].

The properties of the final bigel depend on the process parameters and the individual properties of the hydrogel and the oleogel, as well as on their mixing ratio [155]. The advantages of bigel over emulsions, hydrogels and oleogels include the ease of preparation through simple mixing, stability at room temperature, easy structural formation, the ability to simultaneously encapsulate and deliver both hydrophilic and lipophilic compounds, as well as prebiotics and probiotics microorganisms, and the ability to control the release of the encapsulated substances. Their disadvantages include potential instability at higher temperatures, since most of them are not thermoreversible, their limited application in food systems, and the difficulty in forming a homogeneous gel when the oleogel or hydrogel have high viscosity.

Bigels can be categorized into three main types based on their phase structure. The first type is oleogel-in-hydrogel, where the lipid phase (oleogel) is dispersed within the continuous aqueous phase (hydrogel). The second type is hydrogel-in-oleogel, in which the hydrogel represents the dispersed phase, while the oleogel forms the continuous matrix. The third type is referred to bi-continuous, a more complex hybrid gel system in which it is not clearly defined which phase is continuous and which is dispersed, as both gels form interpenetrating networks [154,157].

The process parameters that significantly affect the final characteristics of bigels have been extensively discussed in other reviews [9,155,156,158]. The following section focuses on the formulations, properties, and applications of bigels formed using waxes, as reported in the literature from 2018 to the present (May 2025).

**Table 3:** Overview of studies on wax-based bigels for food applications (from 2018 to the present).

Oleogel		Hydrogel	OG:HG Ratio	Characteristics Studied and Application	Ref.
Oleogelator	Edible Oil	Hydrogelator			
**Beeswax**					
BW (2%)	Algae oil	Gellan gum (2%)	20:80, 40:60, 50:50, 60:40, 80:20	PLM, CLSM, RM, TPA, FTIR, XRD, NMR, 3D-PA 3D printing	[159]
BW (10%)	Soybean oil	Polyglycerol polyricinoleate (3%)	20:80, 40:60, 50:50, 60:40, 80:20	PS, OM, PLM, CLSM, RM, TPA, FTIR, 3D-PA, 3D printing	[160]
BW (5%)	Canola oil	Sweet potato starch (10%) or Chayote tuber starch (10%)	30:70, 40:60, 50:50	Microscopy, RM, TPA, LBC (oil), XRD, FTIR	[161]
BW (5%)	Sunflower oil	Gel (10%), XG (1%), Agar (15%)	5:95, 10:90, 20:80	PS, BLM, SEM, RM, TPA, FTIR, 3D-PA 3D printing	[162]
BW (20%)	Grape seed oil	SA (2%)	99:1, 95:5, 90:10	PLM, TPA, RM, DSC, XRD, SFC, OS (PV), SE Compound chocolate	[163]
BW (10%), GMS (2%)	Soybean oil	Gellan gum (3%)	30:70, 60:40, 65:35, 70:30, 80:20	PLM, CLSM, RM, FTIR, SS, 3D printing-Prepared foams	[164]
BW (10%)	Sesame oil	SA (3%), Whey protein (25%)	Not reported	FAC, PLM, FTIR, OS (PV, RA, DPPH), Microbiological characteristics, Chemical analysis, SE Cinnamon oil and probiotic strains, -Butter spread	[165]
BW (6%)	Sunflower oil	Tapioca starch (5–10%)	25:75, 40:60, 50:50, 60:40, 75:25	PLM, RM, TPA, DSC, FTIR, SB, in vitro digestion, particle size	[166]
BW (0–3%) + SL (0–1%)	Soybean oil	Flaxseed gum (1%)	90:10 to 30:70	RM, DSC, FTIR, CLSM, probiotic viability, in vitro digestion, FFA Encapsulated probiotics	[167]
BW (0–12%) + DGs (5%)	Soybean DAG oil	Hydroxypropyl methyl cellulose (2%)	50:50	OM, CLSM, DSD, TPA, RM, DSC, FTIR, Product properties-Bread	[168]
BW (8–12%)	Soybean oil	Cellulose nanofibres (2%)	30:70, 40:60, 50:50, 60:40, 70:30	PS, CLSM, LBC (oil), RM	[169]
BW (3%, 6%)	Μedium chain triglycerides oil	SA (2%)	50:50, 80:20, 90:10, 95:5, 99:1	BFM, TPA, RM, XRD	[170]
BW (5%)	Sunflower oil	Agar (15%) or Gel (10%), + XA (2%)	20:80, 30:70, 40:60	RM, TPA, 3D-printing	[171]
BW (12%) + Plant Sterol Esters (8%)	Diacylglycerol corn oil	Gel (5%) + Whey protein isolate (5%)	20:80 to 80:20	PS, CM, OM, PLM, CLSM, TPA, DSC, FTIR, XRD, SE, LBC (oil and water), OS (PV, TBA)	[172]
BW (20%)	Corn oil	Soy protein isolate (20%)	5:95, 10:90, 15:85, 20:80, 25:75, 30:70	CLSM, SEM, RM, NMR, XRD, 3D printing	[173]
BW (15%)	Corn oil	κC + XG (1:1) (1.5%)	20:80, 30:70, 50:50, 70:30, 80:20	CLSM, PLM, RM, XRD, 3D printing	[174]
BW (10%)	Canola oil	SA or carboxymethylcellulose (3%)	50:50	FAC, OS (PV, AV), CLSM, TPA, RM, XRD, LBC, Product properties-Cookies	[175]
BW (4–8%), GMS (4–8%)	Corn oil	κC (0.75%) and Tween 20 (0.5%)	50:50	CLSM, DSD, RM, TPA, DSC, XRD, FTS	[176]
BW (6%) + glyceride monooleate (2%)	Sunflower oil	Agar (0.5–2%)	90:10, 80:20, 70:30, 60:40	RM, CLSM, UV/Vis, stability of bigel films BG films for fresh meat	[177]
BW (10%)	Corn germ oil	Myofibrillar protein	10:90, 30:70, 50:50, 70:30, 90:10	CLSM, TPA, RM, DSC, FTIR, XRD	[178]
BW (20%)	Soybean oil	κC (2%) + starch (10%)	75:25, 50:50, 25:75	OM, RM, TPA, DSC, FTIR, SB, FTS	[179]
BW (1%) + GMS (1%), + lycopene (0.1%)	Soybean oil	Gellan gum (0.3%)	10:90 to 60:40 (*w*/*w*)	CLSM, TPA, RM, DSC, FTIR, SB, in vitro lycopene release profile Designed for lycopene encapsulation and controlled release	[180]
**Carnauba wax**					
CBW (10%) + SL (0.5–1.5%)	Sunflower and olive pomace oil	Gel (5%), Col (15.6%), Agar (2.5%), and combinations	60:40	CM, TA, RM, LBC (water and oil), OS (PV), AC Lingonberry pomace, Edible spreads	[181]
CBW (9.3%) + SL (0.6%)	Sunflower and olive pomace oil	Col (40, 60%)	40:60, 50:50, 60:40	CM, TPA, RM Dysphagia product	[182]
CBW (15%) + SL (2%), + chlorophyll extract (2 types)	Sunflower oil	Agar (5%) + XG (1%)	80:20, 60:40, 40:60, 20:80	PS, CM, CLSM, RM, FTIR, LBC, DA, 3D-printing	[183]
CBW (7%)	Canola oil, Thyme essential oil (0.5–2%)	CPI (15%), microbial transglutaminase	50:50, 40:60, 30:70, 20:80, 10:90	CM, PLM, RM, FTIR, DSC, LBC (water and oil), AC	[184]
CBW (8%)	Canola oil	AG (4%)	90:10	OM, CLSM, TA, RM, DSC, FTIR, XRD, LBC, OS	[185]
CBW (10%)	Rice bran oil	ιC (3%)	40:60, 50:50, 60:40	PS, CM, TPA, RM, DSC, FTIR, XRD 3D-printing	[186]
CBW (9%)	Sunflower oil and olive pomace oil	Col (60%) + SDF (from cranberry and sea buckthorn berry pomace) (1.34%)	25:75	PS, RM, Viability of probiotic cells, Product properties Encapsulation probiotics, Butter spread	[187]
**Candelilla wax**					
CDW (5%)	Canola oil	GCS (5%)	20:80, 40:60, 60:40	OM, RM, FTIR	[109]
CDW (5%)	Canola oil	GCS (5%)	50:50	Dough and cookies properties Shortening substitute for cookies	[52]
CDW (7.5%)	Soybean oil	Egg whites (5–10%)	80:20, 60:40, 40:60, 20:80	CLSM, PLM, RM, TPA, DSC, Emulsions Stability	[188]
CDW (15%), MGs (15%), CDW + MGs (7.5 + 7.5%)	Olive, sunflower, sesame, and soybean oil	Agar (1–4%), κC (0.5–2%), and combinations of them	80:20, 60:40, 40:60, 20:80	CM, OM, PLM, DSD, TPA, DSC, FTIR	[189]
CDW (5%)	Corn oil	Potato protein isolate (15–25%)	30:70, 10:90	CLSM, RM, TPA, DSC, in vitro digestion Encapsulated curcumin	[190]
CDW (4%)	Walnut oil	Potato starch (3.3%)	2:1, 1:1, 1:2, 1:3, 1:4	SFC, FTIR, XRD, NMR Margarine	[191]
GMS (10%) + CDW (2–8%), Paprika oleoresin (0.3%)	Canola oil	Guar gum (0.5%)	2:8	Encapsulation efficiency, CLSM, PO released, CM, RM Phenoxyethanol (0.55%) or caprylyl glycol (0.45%) addition	[192]
γ-β (8%, 3/2), MGs (8%), or CDW (8%) + span 65 (0.7%)	Walnut oil	Chitosan, Sodium tripolyphosphate	40:60, 50:50, 60:40, 70:30, 80:20	SEM, CLSM, RM, TPA, DSC, FTIR, XRD, LBC, SFC, SE Spread replacement	[193]
CDW (6%)	Canola oil	Puratein C (15%)	30:70, 40:60, 50:50, 60:40	Protein characterization, CHNS elemental analysis, LSM, TPA, RM, FTIR, DSC, TGA Transglutaminase (0–35%)	[194]
CDW (8%) + Sucrose ester (0.66%)	Canola oil	XG (0.5%)	55:45, 65:35, 75:25, 85:15	CLSM, RM, TPA, DSC, LBC	[195]
CDW (3%) + MGs or SL (1%)	High oleic acid sunflower seed oil	Fish Gel (5%)	30:70, 50:50, 70:30	PLM, RM, TPA, 3D-PA 3D-printing	[196]
CDW (3%) + MGs or SL (1%)	High oleic acid sunflower seed oil	Fish Gel (5%)	30:70, 50:50, 70:30	CSLM, XRD, FTIR, NMR, 3D-PA, in vitro digestion, HPLC Encapsulated with catechin (0.1% in HG) and quercetin (0.1% in OG)	[197]
**Rice bran wax**					
RBW (7.5%)	Soybean oil	Gel (10%)	40:60, 50:50, 60:40, 70:30	PS, CLSM, RM, DSC, LBC (oil and water), OS (PV), FTS	[198]
RBW (2%) + GMS (1%)	Corn oil	SA (2%)	60:40, 50:50, 40:60, 30:70, 20:80, 10:90	BFM, RM, TPA, Product properties-Dough and baked bread	[199]
RBW (7.5%)	Soybean oil	Gel (7–8%)	70:30, 60:40	Sausage properties Fat replacement in sausage	[200]
RBW (9%) + MGs (0–2%)	Soybean oil	SA (1%) + κC (0.5%)	70:30, 80:20	CLSM, RM, DSC, LBC, NMR	[201]
RBW (10%)	Soybean oil	Gel (5–10%)	50:50, 40:60, 30:70, 20:80	CSLM, RM, DSC, FTIR	[202]
RBW (10%) + DGs (0–3%)	Soybean oil	Gel (7%)	60:40, 70:30, 80:20	CSLM, TPA, FTIR, NMR, LBC	[203]
RBW (8%, 9%)	Walnut oil	Guar gum (1.8%)	30:70, 50:50, 70:30	OM, RM, TPA, FTIR, OBC, Propyl paraben (0.02% *w*/*w*) (antimicrobial)	[204]
RBW (1–7%)	Sunflower oil	Pea protein (4%) and carboxymethyl cellulose (0.3%)	75:25	CLSM, RM, TPA, FTS Transglutaminase (0.1–0.4%)	[205]
**Sunflower wax**					
SW (6–12%), SW + MGs (6–12%)	Olive oil	Agar (2%) + κC (1%)	80:20, 60:40, 40:60, 20:80	CM, OM, PLM, DSD, DSC, TPA, FTIR, SB, LBC (water and oil), OS (PV)	[206]
SW (5%)	Soybean oil	Spirulina platensis protein (1%) + XG (1%)	20:80, 40:60, 50:50, 54:46, 56:44, 58:42, 60:40, 80:20	OM, PLM, CLSM, RM, TPA, FTIR, 3D-PA, 3D-printing	[207]
**Different/combined waxes**					
CDW, CBW, RBW, BW (12–20%) + MGs (1%)	Canola oil	XG (1%)	80:20	PLM, TPA, SFC, Product properties-Croissant preparation	[208]
BW (6%) + RBW (4%)	Soybean oil	Gel (10%)	80:20, 60:40, 50:50, 40:60, 20:80	OM, RM, TPA, DSC, FTIR, FTS	[209]

SW: sunflower wax, CBW: carnauba wax, CDW: candelilla wax, BW: beeswax, RBW: rice bran wax, MGs: monoglycerides, XG: xanthan gum, AG: Arabic gum, κC: κ-carrageenan, ιC: ι-carrageenan, Gel: gelatin, Col: collagen, CPI: chicken protein isolate, SDF: soluble dietary fiber, GCS: gelatinized corn starch, PPI: potato protein isolate, γ-β: γ-oryzanol/β-sitosterol, GMS: glyceryl monostearate, SA: sodium alginate, PS: physical stability, CM: color measurement, OM: optical microscopy, PLM: polarized light microscopy, CLSM: confocal laser scanning microscopy, Cryo-SEM: cryogenic scanning electron microscopy, LSM: laser scanning microscope, BLM: bright-field microscopy, DSD: droplet size determination, DSC: differential scanning calorimetry, TGA: thermogravimetric analysis, FTIR: Fourier-transform infrared spectroscopy, RM: rheology measurement, TPA: texture profile analysis, NMR: nuclear magnetic resonance, XRD: X-ray diffraction, 3D-PA: 3D printing ability, DA: decorating ability, SB: swelling behavior, LBC: liquid binding capacity, SS: storage stability, OS: oxidative stability, PV: peroxide values, TBA: determination of 2-thiobarbituric acid, RA: rancimat analysis, DPPH: radical scavenging capacity, AC: *p*-anisidine value, AC: antioxidant capacity, FTS: freeze–thaw stability, DH: determination of humidity, SFC: solid fat content, FAC: fatty acid composition, SE: sensory evaluation.

### 3.2. Composition Design of Wax-Based Bigels

The formulation of bigels is strongly influenced by the selection and combination of components within both the oleogel (OG) and hydrogel (HG) phases. At the core of the oleogel phase is the choice of natural waxes, which act as structuring agents and largely determine the crystallization behavior, network formation, and thermal properties of the final system.

Among the waxes, the most commonly used for bigel formation is BW, known for its excellent oil-binding and viscoelastic properties, and it accounts for approximately 41% of the relevant studies (Figure 1). The next most frequently used wax is CDW, comprising 23% of the studies, followed by RBW (10 studies) and CBW (8 studies). Additionally, SW is gaining attention for its ability to form strong crystalline networks at relatively low concentrations; however, relative studies are limited [206,207] (Table 3).

The concentration used in most studies is relatively low (<10%) compared to other structuring agents, such as monoglycerides (MGs). Among the waxes, BW is used at the highest concentrations (up to 20%), while SW is used at the lowest (as low as 5%). Specifically, the concentration range used for CBW is 7–15% w/w, averaging around 10%. The corresponding concentration range is 3–15% for CDW, 2–10% for RBW, and 5–12% for SW. However, in most studies investigating different concentrations, the primary objective is to explore the lowest effective concentration of gelators in both the oil and aqueous phases. This reduction in wax concentration is often achieved by their combination with co-structuring agents, such as MGs and soy lecithin (SL), to enhance the strength and stability of the oleogel phase. More specifically, MGs have been combined with CDW [189,193,196], RBW [201], SW [206], and different waxes [208]. Diacylglycerols (DGs) have been used with BW [168] and RBW [203], while SL have been combined with CBW [182,183,196] and BW [167]. Binary gelator systems involving glycerol monostearate (GMS) with CDW [192], BW [164,176,180], and RBW [199] have also been reported. Other combinations such as γ-oryzanol/β-sitosterol (γ-β) with CDW [193], Span 65 with CDW [193], sucrose ester with CDW [195], plant sterol esters with BW [172] have also been investigated. Finally, the combination of two different waxes, namely RBW and BW, with gelatin hydrogel has also been studied [209].

The addition of emulsifiers and surfactants, such as SL, GMS, or synthetic agents like Span 65, can significantly affect the stability, microstructure, and encapsulation efficiency of bigels. For instance, SL combined with BW has been shown to improve the survival of probiotic cells during digestion and storage [167], while the addition of MGs has enhanced the interfacial compatibility between the oil and aqueous phases in bi-continuous systems [203].

#### 3.2.1. Edible Oils

The edible oils used for the formation of the oleogel phase vary depending on the desired nutritional profile, oxidation stability, and availability. Soybean oil is the most widely used oil [160,188,203,207,209] due to its neutrality and widespread accessibility, but sunflower oil [65,183,196], olive oil [189,206], corn oil [173,190,199], canola oil [109,161,185,195,208], and walnut oil [191,193,204] are also commonly employed. In addition, rice bran oil [186], algae oil [159], grape seed oil [163], and sesame oil [189,210] have also been studied. Giannakaki et al. [189] investigated the characteristics of bigels using different edible oils and concluded that the type of oil influences the gel’s firmness and oil-binding behavior and may also play a role in the delivery of bioactives or flavor compounds. For example, walnut oil has been used to enhance the nutritional value of bigels intended for functional spreads [193], while olive pomace oil contributes a sustainable and phenolic-rich lipid base [182,187]. Moreover, thyme essential oil has been combined with canola oil in order to provide antioxidant properties [184].

#### 3.2.2. Hydrogelators

The hydrogel phase is typically composed of hydrocolloids, i.e., proteins and polysaccharides, that structure the aqueous matrix and can affect the rheological and mechanical behavior of bigels. Most researchers have used gelatine (Gel) [171,172,181,183,198,200,203,209] to structure the hydrogel phase, while others have utilized agar [177,185], xanthan gum (XG) [171,174,195,208], collagen (Col) [181,182], κ-carrageenan (κC) [189], ι-carrageenan (ιC) [186], sodium alginate (SA) [163,170,175,210], gellan gum [159,164,180], flaxseed gum [167], Tapioca starch [166], guar gum [192,204], fish gelatine [197], gelatinized corn starch (GCS) [109], either alone or in binary mixtures. Several studies have investigated blends of hydrocolloids to improve the structural integrity of the hydrogel phase. Examples include agar + XG [171,183], agar + κC [189,206], SA + κC, agar or gelatine with collagen [181], gelatin + whey protein isolate [172], κC + XG [174], or κC + starch [179]. Additionally, bigels formulated with myofibrillar protein [178], whey proteins isolate [172], and whey protein concentrate (WPC) [210] have also been reported. The use of plant-derived proteins, such as soy or pea protein isolates, is also gaining ground for applications in plant-based or vegan formulations [173,190,194,205].

#### 3.2.3. Oleogel-to-Hydrogel Ratio

The oleogel-to-hydrogel ratio (OG:HG) is a critical design parameter that directly influences the mechanical properties, phase distribution, and application potential of bigels. Ratios ranging from 80:20 to 20:80 have been extensively studied [159,160,174,183,188,206,207,209], with some formulations exhibiting phase inversion depending on the composition and proportion of each phase. Specifically, Pang et al. [172] reported that when the oleogel phase, composed of BW and plant sterol esters, increased from 20% to 80%, the bigel structure transitioned from an oleogel-in-hydrogel to a hydrogel-in-oleogel system, with phase transition occurring between 50% and 60% oleogel content. Similarly, Guo et al. [207] observed a bi-continuous structure at 56–58% SW-based oleogels, with coexisting oil-in-water (O/W) to water-in-oil (W/O) regions. As the OG:HG ratio increased from 54:46 to 60:40, the bigels transitioned from O/W to W/O types. Bigels with higher oleogel content tend to display better oil retention and mimic butter-like textures [199], whereas hydrogel-rich systems often demonstrate higher hardness [206], a characteristic that supports the replacement of animal fat in various meat products. Furthermore, a balanced 50:50 ratio has been shown to offer an optimal compromise between mechanical strength, thermal stability, and printability, particularly in formulations designed for 3D-printing [159,207]. This ratio has also been found effective in preserving the viability and enhancing the delivery performance of probiotic *Lactobacillus plantarum*, when encapsulated within a bigel system composed of a lecithin–BW oleogel and a flaxseed gum hydrogel [167].

### 3.3. Physicochemical and Structural Characterization of Bigels

The physicochemical and structural properties of bigels are mainly influenced by the gelators selected for each phase, their combination with other structurants, the type of oil used in the oleogel phase, and the ratio between the two phases. These properties are also directly affected by the type of bigel formed, namely hydrogel-in-oleogel, oleogel-in-hydrogel, or a bi-continuous. The characterization of the functional properties of wax-based bigels is critical for evaluating their suitability and effectiveness across a wide range of applications, including their use as fat replacers, encapsulation matrices for bioactive compounds, and even as innovative 3D-printable systems. These functional properties cover several critical aspects, such as mechanical strength, viscoelastic behavior, phase stability under various storage and processing conditions, and the ability to protect and control the release of encapsulated compounds.

Specifically, the characterization typically involves a combination of techniques to comprehensively assess the system. Visual analysis and physical evaluations, such as inversion tests, visual assessment, and color measurements, provide initial insights into uniformity and stability. Microstructural analysis using various microscopy techniques, including PLM and Cryo-SEM, helps to reveal the internal architecture and phase distribution. Rheological and textural studies, through rheometry and texture profile analysis (TPA), as well as DSC, thermogravimetric analysis (TGA), FTIR, XRD are also used to explore texture, thermal and other properties. Additionally, the evaluation often extends to studies of release profiles, swelling behavior, and liquid binding capacity to understand functional performance in targeted applications. Storage stability tests, including assessments of oxidative stability and freeze–thaw resistance, further ensure that these bigels maintain their desired properties over time. Altogether, this multifaceted characterization is crucial for tailoring wax-based bigels to meet specific demands in food, nutraceutical, and emerging technological applications.

#### 3.3.1. Physical and Sensory Properties

The evaluation of the physical properties of wax-based bigels is a crucial first step toward assessing and optimizing their functionality for specific applications. Physical and sensory attributes such as appearance, homogeneity, and color are directly linked to product stability, consumer acceptance, and overall performance, particularly when these systems are intended for use in food products. Visual assessments and inversion tests are commonly used to quickly and effectively evaluate the uniformity and self-standing ability of bigels, aiding in the selection of appropriate concentrations and process conditions [201,203,206]. Most researchers report that bigel systems are self-stable across a wide range of mixing ratios and storage conditions; however, Saffold and Acevedo [203], Nutter et al. [201], and Giannakaki et al. [189] observed phase separation in systems with OG:HG ratios of 30:70 and 40:60.

The color of bigels is affected by several factors, including the type and concentration of gelling agents, the oil used in the oleogel phase, and the oleogel-to-hydrogel ratio. Dimakopoulou-Papazoglou et al. [206] found that all bigels composed of SW, MGs, agar, and gelatin were opaque and light yellow in color, while Oyom et al. [184] reported that increasing the hydrogel content in bigels resulted in lighter-colored gels. Color is a critical parameter assessed during bigel formation, as it plays an important role in ensuring consumer acceptance when these systems are incorporated into various food applications.

#### 3.3.2. Microstructure Analysis

Microscopy examination is one of the primary techniques used to characterize bigels, as it explores the microstructure of the system and the distribution of phases, providing valuable insights into how the oleogel and hydrogel networks coexist and interact within the matrix. Most researchers employ optical microscopy (OM) [109,179,204,209], PLM [163,172,196,206,207,208], bright-field microscopy (BLM) [162,170,199], and confocal laser scanning microscopy (CLSM) [159,164,167,168,198,201,203], while some also utilize cryogenic scanning electron microscopy (cryo-SEM) [167,173]. These techniques are commonly used to characterize the individual phases and to determine when the system transitions from an oil-in-water (O/W) structure to a water-in-oil (W/O) structure, or when a bi-continuous phase is formed. For example, Chao et al. [159] reported that at OG:HG ratios of 20:80 and 40:60, the system exhibited an OG-in-HG structure; at a 50:50 ratio, a bi-continuous structure was observed; and at ratios of 60:40 and 80:20, an HG-in-OG structure was present. As also discussed earlier in the section on oleogels, the type and concentration of gelators, the oil used, and the oleogel-to-hydrogel ratio influence the formation of different crystal morphologies and the size of oleogel and hydrogel droplets. In general, it can be stated that these properties vary depending on the specific systems studied, highlighting the complexity and versatility of bigel microstructures.

Additionally, several researchers have used microscopy images to perform droplet size determination (DSD) [168,176,189,206]. For example, Yang et al. [176] observed that increasing the BW content from 4% to 8% led to a reduction in droplet size from 56.11 μm to 31.88 μm. Similar results were reported by Dimakopoulou-Papazoglou et al. [206], where increasing the SW concentration from 6% to 12% reduced the size of lipid droplets from 36.9 μm to 27.6 μm in 20:80 (OG:HG) bigels. Moreover, the addition of MGs to the oleogel phase resulted in a more uniform distribution of lipid droplets, and as the total concentration in the system increased, the droplet size further decreased.

#### 3.3.3. Rheological and Textural Properties

Rheological and textural analyses are essential for evaluating the mechanical behavior and structural integrity of bigels, which directly impact their applicability in food products. Texture profile analysis (TPA) assesses parameters such as hardness, cohesiveness, chewiness, and springiness, offering a quantitative understanding of textural characteristics of bigels. Most researchers evaluate bigels using either rheometry, texture analysis, or a combination of both techniques, as summarized in Table 3. These analyses are critical for optimizing formulations, processing parameters, and storage conditions, as well as for ensuring the desired functional properties and consumer acceptance when bigels are used as fat substitutes, delivery systems, or novel structured materials. The specific properties required of bigels vary depending on their intended application. For example, bigels designed to replace fat in sausages need to have higher hardness [200], whereas those formulated for spreads [181,191,193] or bakery products [168,175,208] generally require softer structures.

Chao et al. [159] reported that the hardness of BW/gellan gum bigels increased with higher proportions of the hydrogel phase. Vershlov & Davidovich-Pinhas [195] found that bigels stored at 4 °C exhibited greater firmness, cohesiveness, and plasticity compared to those stored at 25 °C, with the best mechanical behavior observed at a 65:35 oleogel-to-hydrogel (OG:HG) ratio. Similarly, Giannakaki et al. [189] showed that hardness increased with higher κC and CDW content, with bigels containing 15% CDW exhibiting the highest hardness and chewiness. In contrast, MG-based systems displayed lower hardness due to the presence of larger droplets, which disrupted the hydrogel network.

#### 3.3.4. Thermal Properties

Thermal analysis has been employed to investigate melting and crystallization profiles, phase transitions, and the thermal stability of the oleogel and hydrogel networks within bigels. DSC has shown that bigels exhibit distinct thermal behaviors influenced by the characteristics and ratios of their constituent phases. Multiple studies have consistently demonstrated that bigels retain the thermal properties of their individual oleogel and hydrogel phases without forming new thermal transitions, indicating physical rather than chemical interactions [189,190,206]. Saffold and Acevedo [202] observed distinct dual endothermic peaks in DSC thermograms, corresponding to the melting of each gel phase, while higher oleogel content was associated with improved thermal stability. Zhu et al. [180] reported that bigel formulates with gellan gum hydrogel and GMS-BW oleogel exhibited a higher melting point (~50 °C) compared to oleogels (~46 °C), highlighting the stabilizing effect of the hydrogel. Similarly, Pang et al. [2] found that bigels had higher melting temperatures than individual oleogels or hydrogels, as DSC indicated multiphase melting behavior resulting from different water states and lipid polymorphism. Vershlov and Davidovich-Pinhas [195] noted that the melting behavior of bigels closely followed that of the wax phase, with minor influence from hydrogel content.

#### 3.3.5. Fourier Transform Infrared Spectroscopy (FTIR) and X-Ray Diffraction (XRD)

FTIR is widely utilized in the analysis of bigels as it enables the identification of specific chemical bonds and the assessment of interactions between the components of the oleogel and hydrogel phases. The peaks observed in the FTIR spectra, typically in the range of 4000–400 cm^−1^, are linked to the gelators used to structure the two phases, their interactions, as well as the oil used in the system. Additionally, the oleogel-to-hydrogel ratio significantly influences the FTIR spectra, leading to changes in the intensity of peaks associated with hydrogen bonding and other functional groups [197,206,207]. For instance, systems with a higher hydrogel content show increased intensity in the broad band around 3700–3100 cm^−1^, which corresponds to O–H stretching vibrations, indicating the presence of hydrogen bonds [160,178,202]. In contrast, as the proportion of the oleogel phase increases, the intensity of the characteristic peaks of oleogels increases, such as those at approximately 2918, 2850, and 1465 cm^−1^, corresponding to C–H stretching vibrations, and at 1743 cm^−1^, related to the stretching vibrations of carbonyl groups (C = O) from glycerol and fatty acids, appear with higher intensity [159,178]. Overall, studies consistently show that no new peaks appear in the spectra, suggesting that no new chemical bonds are formed between the oleogel and hydrogel phases and the bigel formation is mainly governed by physical interactions [172,178,189,202,207].

XRD has provided valuable insights into the crystalline characteristics of bigels, highlighting the influence of both the type and proportion of oleogelators used [176]. Ghorghi et al. [163] observed minimal variation in the XRD patterns of bigels formulated with BW and SA in different oleogel-to-hydrogel ratios, indicating that the crystalline structure was primarily determined by the oleogel phase. Xie et al. [197] reported that increasing the oleogel content in CDW-based bigels led to higher crystallinity, with XRD patterns dominated by peaks corresponding to β and β′ crystal forms. Similarly, Pang et al. [172] identified α, β, and β′ polymorphs in bigels, with β′ forms being particularly prominent in water-in-oil systems. Additionally, Quilaqueo et al. [175] reported that bigels, made with BW, SA and carboxymethylcellulose, were semi-crystalline solids composed of both crystalline and amorphous regions. In contrast, Martins et al. [170] observed no polymorphism in BW/SA bigels, regardless of the oleogel-to-hydrogel ratio.

#### 3.3.6. Swelling Behavior

The swelling behavior reflects the ability of the system to absorb water, which is closely linked to its structure and functionality. This characteristic depends almost exclusively on the hydrogel phase and it directly affects the potential applications of bigels in various food products, as well as the release of encapsulated bioactive compounds. Li et al. [166] observed that the OG:HG ratio and the type of bigel significantly affected swelling behavior, with 40:60 (BW/Tapioca starch) bigels exhibiting the highest water absorption (27.4%). The swelling ratio decreased as the oleogel content increased, due to the hydrophobic nature of the oleogel phase [179,180,206]. Moreover, the choice of gelling agents also impacts water uptake. Li et al. [166] and Zhou et al. [179] reported that starch enhanced swelling due to its hydrophilic properties, which facilitated water absorption in the hydrogel phase. The addition of MGs led to increased water absorption, even at a 20:80 ratio, attributed to their emulsifying capacity and hydrophilic nature [206]. According to Dimakopoulou-Papazoglou et al. [206], bigels composed of agar, carrageenan, SW, and MGs exhibited very low swelling values (0.3–3.4%). Finally, it is worth noting that, as reported by Zhu et al. [180], the release of lycopene was slower in bigels with higher oleogel fractions after complete digestion, highlighting their potential for the controlled release of encapsulated compounds.

#### 3.3.7. Liquid Binding Capacity

The liquid binding capacity (LBC) is evaluated as it provides insights into the system’s ability to retain solvents, both water and oil, within the different phases of the bigel matrix. LBC is typically determined by measuring the amount of liquid released after applying centrifugation (e.g., 5000–10,000 rpm for 15 min) [172,181,184,195] at room temperature, although it can also be assessed by applying pressure on the sample (e.g., 1 kg for 10 min on cubes) [206]. Vershlov & Davidovich-Pinhas [195], who studied bigels composed of CDW, sucrose ester, and xanthan gum at various OG:HG ratios, observed that the samples maintained over 99% oil binding capacity (OBC) during storage at both 4 °C and 25 °C. Additionally, Oyom et al. [184] reported that a 50:50 ratio resulted in the lowest levels of water and oil release. The composition of the bigel directly influences the LBC, as noted by Baltuonyte et al. [181], who found that bigels containing agar and collagen formed stronger gel networks and therefore exhibited lower total liquid release compared to gelatin-based systems. Pang et al. [172] reported that a 60:40 bigel maintained a very high capacity to retain water and oil even after 30 days of storage. Similar findings were reported by Liu et al. [169], who evaluated bigels containing BW and cellulose nanofibers for storage stability over 28 days. These researchers concluded that such stability highlights the potential applications of bigels in the food industry, especially when used as fat replacers or delivery systems.

#### 3.3.8. Storage Stability

##### Oxidative Stability

The development of oxidation of bigels is influenced by multiple factors, including the oil composition, the oleogel-to-hydrogel ratio, processing conditions such as time and temperature, storage conditions, and the use of antioxidants. The oxidative stability of bigels is monitored by PV [163,181,198,206,210], TBARs and *p*-anisidine value (AV) [172,175]. Dimakopoulou-Papazoglou et al. [206] reported that bigels (formulated with SW, MGs, agar, and κ-carrageenan) at different OG:HG ratios exhibited greater oxidative stability than liquid olive oil but were less stable than oleogels, with the PV values of the bigels remaining within acceptable limits up to day 35 when stored at 5 °C. In contrast, Cho et al. [198] observed very low PV values (<3 meq/kg) during ambient storage of bigels made with RBW and gelatin for over six months, even without the addition of antioxidants. Additionally, Quilaqueo et al. [175] found that AVs were significantly lower in bigels composed of either BW with sodium alginate (SA) or BW with carboxymethylcellulose at a 50:50 ratio compared to BW oleogels, suggesting that the aqueous phase can protect against oxidation and that the choice of hydrogelator plays a critical role. It is also noteworthy that Oyom et al. [184] observed that incorporating thyme essential oil significantly enhanced the antioxidant capacity of all gel types (CBW with chickpea protein isolate, CPI), even at low concentrations (0.5%), by improving radical scavenging activity.

##### Freeze–Thaw Stability

The freeze–thaw stability of bigels refers to their ability to maintain structural integrity, homogeneity, and functional properties after undergoing repeated cycles of freezing and thawing. This parameter is critical for assessing the suitability of bigels in frozen food applications and is typically evaluated by measuring oil (OHC) and water (WHC) holding capacity after freeze–thaw cycles. A high freeze–thaw stability indicates minimal phase separation, reduced oil or water leakage, and preservation of textural and rheological characteristics, thereby ensuring product quality throughout its shelf life. Zhou et al. [209] reported that bigels, formulated with BW, RBW and gelatin, maintained high oil retention after three freeze–thaw cycles, with BG 40:60 retaining approximately 80.5% OHC and BG 20:80 about 90.9%. The authors attributed this to the protective matrix formed by gelatin, which effectively resisted oil leakage. Among the formulations, BG 40:60 exhibited a bicontinuous structure with superior viscoelastic and freeze–thaw properties, suggesting its potential as a low-calorie butter substitute. Similarly, Cho et al. [198] observed that bigels (RBW and gel) exhibited roughly 50% less liquid loss compared to standalone oleogels or hydrogels after freeze–thaw cycles. Notably, the 50:50 and 60:40 formulations showed the lowest total liquid loss and best structural integrity. Furthermore, Zhou et al. [179] demonstrated that bigels formulated with starch possessed enhanced freeze–thaw resistance, maintaining their structure over multiple freeze–thaw cycles. This improved performance was linked to stronger hydrogen bonding interactions between starch and water, which contributed to better oil retention and reduced phase separation.

## 4. Applications of Natural Wax-Based Gelators in Food Systems

Bigels and oleogels structured with natural waxes are gaining momentum as alternatives to traditional fats in food systems. These structuring systems not only help reduce saturated and trans fats but also offer opportunities to incorporate functional ingredients and improve texture. Their unique rheological and thermal properties make them suitable for a wide range of applications, including meat products, baked goods, spreads, and functional foods.

### 4.1. Applications of Natural Wax-Based Bigels

Bigels incorporating natural wax-based oleogels and hydrogels are increasingly being explored as multifunctional systems in food applications. Their unique biphasic structure, combining both lipid and aqueous networks, allows them to act as fat replacers, structuring agents, or carriers for bioactive compounds in a wide range of food formats.

**Figure 2:**
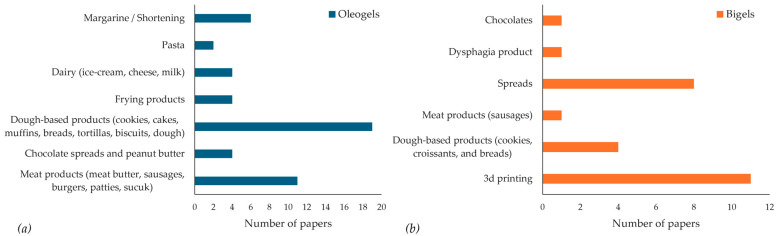
Applications of natural wax-based gels in food systems. (**a**) Number of studies reporting the use of natural wax-based bigels across different food applications; (**b**) Number of studies applying natural wax-based oleogels.

A growing number of studies have demonstrated the suitability of wax-based bigels in 3D food printing, leveraging their tunable rheological and structural properties (Figure 2). Bigels structured with BW, CDW, SW, and CBW, in combination with gelling agents such as gellan gum, xanthan gum, κ-carrageenan, or hydrocolloid blends, have been tailored for extrusion-based printing. These systems offered favorable consistency, shape retention, and compositional flexibility, making them useful in developing personalized or functional foods [158,160,162,164,171,173,174,183,186,196,207].

Several bigel systems have been used to replace conventional fats in baked goods and dough-based products. BW and RBW-based bigels have been tested in cookies, croissants, and breads, offering similar textural and sensory qualities while reducing saturated fat [168,175,199,208]. In particular, studies report good dough machinability, moisture retention, and consumer acceptability.

Bigels have also shown promise in meat product reformulation. In coarse-ground sausages, RBW-based systems have been used to partially or fully replace animal fat, maintaining structural integrity and improving nutritional quality [200].

Another notable application is in spreadable and functional food products, where bigels serve as replacements for butter or margarine. Formulations using CBW and CDW with biopolymers such as chitosan, lecithin, or GCS were found to deliver spread-like texture and appearance, while also accommodating the incorporation of plant-based oils [52,181,187,191,193]. CBW-based bigels with lingonberry pomace extract were also developed as functional edible spreads with antioxidant enrichment [181]. In addition, systems with added dietary fibers and probiotics demonstrated effective fat structuring and encapsulation [187,210].

Bigels with targeted nutritional or clinical applications have been explored for individuals with swallowing difficulties. A dysphagia-suitable bigel formulation with CBW and lecithin produced gels with appropriate consistency and safe swallowing behavior [182]. Other bigels were evaluated for their suitability in compound food systems, such as filled chocolates, where BW-based matrices helped maintain desirable viscosity and melting profiles [163].

### 4.2. Applications of Natural Wax-Based Oleogels

Wax-based oleogels have been widely explored in recent years as functional alternatives to conventional solid fats in diverse food systems (Figure 2). Their tunable structure, textural behavior, and oxidative stability have enabled applications in frying, baking, meat products, spreads, margarine formulations, and plant-based or functional foods.

A major area of application is in frying, where oleogels structured with BW, SW, CBW, or combination of waxes have been evaluated as replacements for conventional frying fats. For instance, SW and MGs-based oleogels were used to fry French fries, offering improved texture and reduced oil uptake [85], while BW-based systems reduced oil absorption and preserved the quality of fried potato strips [56]. Similarly, CBW oleogels have been proposed for frying traditional Indian snacks like Mathri [61], while applications using combination of waxes in deep-fried potato chips were also demonstrated [68].

In baked goods, oleogels have successfully replaced butter, margarine, or shortening in cookies, muffins, cakes, and pastries. Oleogels containing BW or CDW showed promising sensory and structural properties when used in cookies [33,76,91], while CBW systems demonstrated functionality in pastries such as cheese crackers and bow tie cookies [126]. Margarine-like formulations using BW, RBW, or mixed waxes have also been developed with appropriate melting behavior and plasticity [67,108]. CDW-based oleogels were used to prepare sponge cake with canola oil blends [71], and combinations of CDW with stearic acid or olive diacylglycerol stearin were studied for their performance in pasta [127]. Another study explored CDW for replacing saturated fats in sponge cake bread [72]. BW-based oleogels have also been used to formulate gluten-free cakes [110]. Several papers demonstrated the ability of SW, RBW, and mixed wax oleogels to replicate the structural roles of margarine and shortening in cakes and pastries [82,141]. In one study, conventional shortening was replaced with combinations of BW and RBW in safflower oil [82]. In another case, cold-pressed hempseed oil was structured with different waxes to reduce saturated fat in margarines and spreads [141].

In meat products, wax-based oleogels have attracted interest as fat replacers in sausages and burgers. BW and ethyl cellulose-based oleogels using blends of fish, olive, and linseed oils were used to replace pork backfat in liver pâtés and low-fat burgers with positive sensory acceptance [102,111]. RBW systems also enabled reductions in saturated fat content in frankfurters and bologna sausages without compromising texture [55,66,133]. Similarly, CDW and quercetin-enriched systems successfully replaced animal fat in meat batter and sausages [48]. Other studies demonstrated the feasibility of pork backfat replacement using BW-linseed oil oleogels in frankfurters [92] and sausages [113]. RBW and SW oleogels were also evaluated in frankfurters and bologna sausages [55,133]. BW and CDW have additionally been studied as replacements for animal fat in beef burgers and meat patties [89,97]. Finally, sucuk-type fermented sausages were successfully formulated using flaxseed oil oleogels structured with BW and SW [47].

Spreads and confectionery products could also benefit from oleogel technology. Butter in chocolate spreads was replaced with blends of CDW and MGs [39]. CBW-based systems exhibited appropriate thermal and structural behavior in chocolate spreads and cakes [54,125], while imitation cheese made with CBW oleogels maintained desirable texture and melting properties [70]. These systems offered improved spreadability and thermal stability. In addition, CDW oleogels were used as water substitutes in pasta [96], while other formulations targeted dough preparation using blends of BW, RBW, and SW [88]. Another study used CBW, MGs and β-sitosterol blends in sunflower oil for tender doughs such as cookies and pastries [31]. Several studies have also used oleogels for partial or full butter substitution in ice-creams, cream fillings, cakes, muffins, cookies, and composite pastries [30,32,62,80,123,128,129]. Finally, oleogels have been utilized in dough development and snack products. Mixed wax systems containing BW, CBW, and MGs enhanced dough workability and rheology in short-dough biscuits and cookies [87], while applications in maize tortillas were also demonstrated [73].

In functional or plant-based foods, novel wax blends have enabled customized formulations. For example, a plant-based ice cream matrix using CDW in combination with oat milk and millet milk was proposed by [88], while other authors explored hemp seed oil-oleogels or insect oil-based systems as sustainable fat sources in bakery applications [90,142]. RBW and SW oleogels were also evaluated for margarine replacements with reduced saturated fats and better nutritional profiles [122].

Some applications also explored the use of oleogels in cheese and peanut butter. RBW and SW blends were shown to replace solid milk fat in processed cheeses like Swiss cheese [140], and fully hydrogenated cottonseed oil structured with multiple waxes was applied as a stabilizer in peanut butter [21].

## 5. Conclusions and Future Perspectives

In recent years, natural waxes have been extensively studied as structuring agents in oleogels and bigels, offering promising alternatives to conventional solid fats in a variety of food systems. Their ability to form stable gel networks at low concentrations, along with their natural origin and safety status, makes them attractive for food applications aiming to improve nutritional profiles without compromising quality. Many studies have focused on characterizing their physicochemical, thermal, and rheological behavior, as well as evaluating their performance in baked goods, meat products, spreads, and more recently, 3D-printed foods.

Despite this significant progress, there is still ample room for further research and development. The potential of combining different waxes or exploring new mixtures of waxes with other gelators remains largely untapped and could lead to the design of structured systems with improved or tailored functionalities. Likewise, most studies so far have employed a limited set of edible oils. Testing a broader variety of plant-based oils could support the development of more sustainable and application-specific systems.

Moreover, while several food applications have already been explored, there is clear potential to expand into new product categories and processing conditions. For example, the use of wax-based structured oils in dairy alternatives, frozen desserts, fillings, sauces, or emulsified products could open new opportunities in both traditional and plant-based formulations.

## Figures and Tables

**Table 1:** Chemical composition and melting points of natural waxes used in the formation of oleogels and bigels.

Wax	Composition	Melting Point (°C)	Source	Ref.
Beeswax (BW)	Wax esters: 60–80%, Hydrocarbons: 10–25%, Free fatty acid: 10–15%, Free fatty alcohol: 0–5%	61–65	Animal (secretion by honeybees)	[14,15]
Carnauba wax (CBW)	Wax esters: 50–70%, Hydrocarbons: 1.5–3%, Free fatty acid: 3–6%, Free fatty alcohol: 15–30%, Resins/others: 6.5–10%	80–85	Plant (leaves of *Copernicia prunifera*)	[13,16]
Candelilla wax (CDW)	Wax esters: 20–30%, Hydrocarbons: 60–65%, Free fatty acid: 7–10%, Free fatty alcohol: 10–15%	68–73	Plant (stems/leaves of *Euphorbia antisyphilitica*)	[13,14,17]
Rice bran wax (RBW)	Wax esters: 90–97%, Free fatty acid: 3–6%, Resins/others: 3–8%	78–82	Plant (by-product of rice bran oil refining)	[13,14,18]
Sunflower wax (SW)	Wax esters: 96–97%, Free fatty acid: 0–1%, Resins/others: 0–3%	75–80	Plant (sunflower seed oil processing)	[13,14]

## Data Availability

No new data were created or analyzed in this study.
